# Cyanuric Acid Hydrolase from *Azorhizobium caulinodans* ORS 571: Crystal Structure and Insights into a New Class of Ser-Lys Dyad Proteins

**DOI:** 10.1371/journal.pone.0099349

**Published:** 2014-06-10

**Authors:** Seunghee Cho, Ke Shi, Jennifer L. Seffernick, Anthony G. Dodge, Lawrence P. Wackett, Hideki Aihara

**Affiliations:** 1 Department of Biochemistry, Molecular Biology, and Biophysics, University of Minnesota, St. Paul, Minnesota, United States of America; 2 BioTechnology Institute, University of Minnesota, St. Paul, Minnesota, United States of America; 3 Microbial and Plant Genomics Institute, University of Minnesota, St. Paul, Minnesota, United States of America; Universidade Nova de Lisboa, Portugal

## Abstract

Cyanuric acid hydrolase (CAH) catalyzes the hydrolytic ring-opening of cyanuric acid (2,4,6-trihydroxy-1,3,5-triazine), an intermediate in *s*-triazine bacterial degradation and a by-product from disinfection with trichloroisocyanuric acid. In the present study, an X-ray crystal structure of the CAH-barbituric acid inhibitor complex from *Azorhizobium caulinodans* ORS 571 has been determined at 2.7 Å resolution. The CAH protein fold consists of three structurally homologous domains forming a β-barrel-like structure with external α-helices that result in a three-fold symmetry, a dominant feature of the structure and active site that mirrors the three-fold symmetrical shape of the substrate cyanuric acid. The active site structure of CAH is similar to that of the recently determined AtzD with three pairs of active site Ser-Lys dyads. In order to determine the role of each Ser-Lys dyad in catalysis, a mutational study using a highly sensitive, enzyme-coupled assay was conducted. The 10^9^-fold loss of activity by the S226A mutant was at least ten times lower than that of the S79A and S333A mutants. In addition, bioinformatics analysis revealed the Ser226/Lys156 dyad as the only absolutely conserved dyad in the CAH/barbiturase family. These data suggest that Lys156 activates the Ser226 nucleophile which can then attack the substrate carbonyl. Our combination of structural, mutational, and bioinformatics analyses differentiates this study and provides experimental data for mechanistic insights into this unique protein family.

## Introduction

Cyanuric acid, or 2,4,6-trihydroxy-1,3,5-triazine, is an industrially important compound used to make pesticides, dyes, and disinfectants. The latter consist largely of N-chlorinated cyanuric acid derivatives, used for cleaning and swimming pool disinfection. In 2005, 350 million pounds of cyanuric acid were produced industrially for those purposes [1]. Although cyanuric acid alone is not very toxic, coingestion with melamine was the cause of recent poisonings due to adulterated pet food [2], leading to the largest pet food recall in North America and a $24 million settlement.

**Figure 1:**
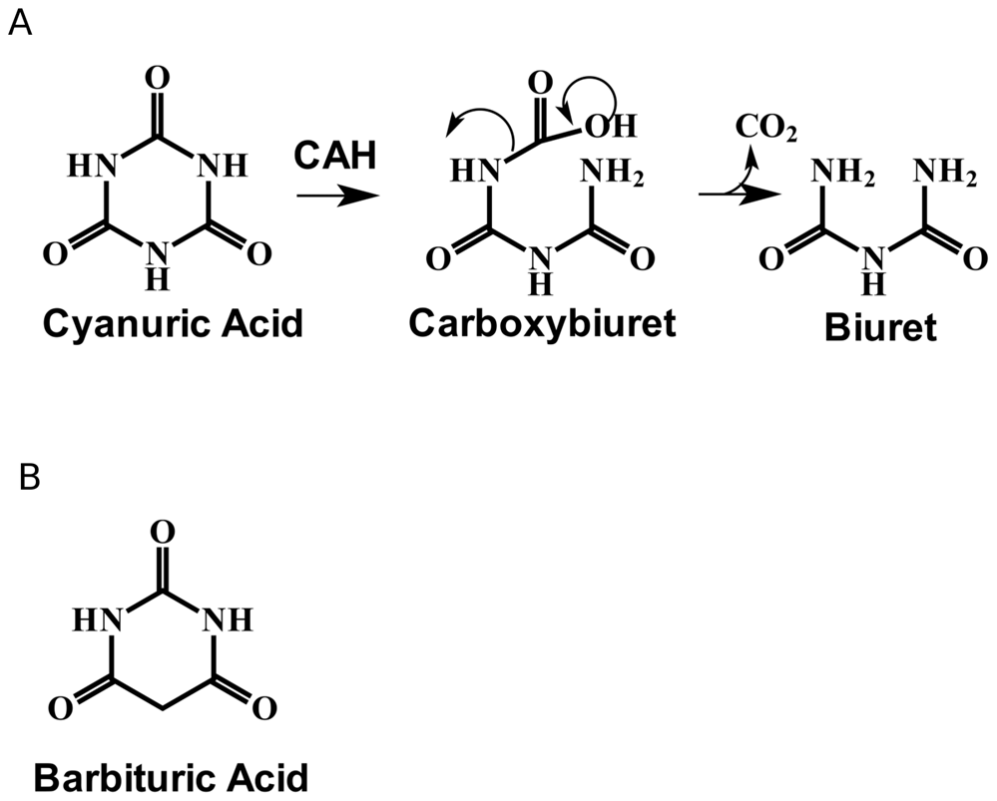
CAH catalytic reaction and inhibitor structure. A. CAH conversion of cyanuric acid to carboxybiuret, which spontaneously decarboxylates to produce biuret. B. Structure of the CAH substrate analog and inhibitor, barbituric acid.

The use of cyanuric acid as a nitrogen source by bacteria generally prevents accumulation in the environment following disinfectant and pesticide degradation [3], [4]. The first step of cyanuric acid metabolism is catalyzed by cyanuric acid hydrolase (CAH) and results in *s*-triazine ring opening [5], [6] to produce unstable carboxybiuret, which undergoes rapid, spontaneous decarboxylation to yield biuret (Fig. 1A) [7]. Biuret is then metabolized by two more enzymes, biuret hydrolase and allophonate hydrolase, resulting in the complete mineralization of cyanuric acid and the release of three nitrogen atoms from the *s*-triazine ring as ammonia.

Recently, bioinformatics analyses determined that cyanuric acid hydrolases were members of an isolated family of enzymes and homologous to barbiturases [7]. This cyanuric acid hydrolases/barbiturase family consisted of only 41 members when initially characterized. Since then, additional cyanuric acid hydrolases have been identified [8], but it still constitutes a unique protein family with no known linkages to other families. Only limited studies have been conducted with barbiturase, a rare enzyme involved in the catabolism of pyrimidines in a limited number of bacteria [9]. Barbiturase is not reactive with cyanuric acid. Conversely, barbituric acid (Fig. 1B) is not reactive with cyanuric acid hydrolases, but it is bound tightly and competitively inhibits the enzyme's activity with cyanuric acid [5], [6]. Since most of the characterized proteins in the cyanuric acid hydrolase/barbiturase protein family react with cyanuric acid and cyanuric acid is not known to be a natural product of any bacterium, plant, or animal, it has been proposed that this might represent a relatively minor protein family, perhaps even a fold that was dying out and has undergone a resurgence with the recent input of commercial *s*-triazine ring compounds into the environment.

In light of the above, there is increasing interest to understand the structure and reaction mechanism of cyanuric acid hydrolases. We have conducted X-ray crystallographic studies of the cyanuric acid hydrolase from *Azorhizobium caulinodans* ORS 571 (NCBI reference sequence; YP_001526808.1), denoted here as CAH. Our preliminary account of the crystallization was previously reported [10]. During the writing of this manuscript, the structure of AtzD, the cyanuric acid hydrolase in *Pseudomonas* sp. strain ADP, was reported [11]. AtzD and CAH are 51% identical in a pairwise amino acid sequence alignment and share an overall unique fold consisting of three structurally analogous domains that form a barrel structure. Unique to the present study are details regarding structural features of subunit contacts and second-tier active site residues, proposed to contribute to catalysis. In addition, the present work developed a highly sensitive, coupled-enzyme assay using biuret hydrolase that allowed the detection of mutant activity diminished by eight orders of magnitude from that of wild-type CAH. This assay was used to differentiate the catalytic roles of the three active site serine residues in CAH which could not be done in the AtzD study. Mutant, bioinformatics, and structural studies within this paper have allowed us to propose a different catalytic serine nucleophile than was previously proposed for AtzD [11].

## Materials and Methods

### Cloning, Site Directed Mutagenesis, Protein Expression and Purification

Cloning the functional CAH gene from *A. caulinodans* ORS 571 [7], and heterologous expression and purification of the encoded native and selenomethionine-labeled proteins [10] were described previously. The protein can be identified by the following reference tags: A8IKD2 (Uniprot), AZC_3892 (locus name), YP_001526808 (accession), and GI158425516. Site directed mutagenesis was conducted with a QuikChange kit (Agilent Technologies, Santa Clara, CA), using the following primers: S79A used 5′-gcctcgtcatggccggcggcacc-3′ and 5′-ggtgccgccggccatgacgaggc-3′, S226A used 5′-gcgcgcgcgagctgtgccagcggt-3′ and 5′-accgctggcacagctcgcgcgcgc-3′, S333A used 5′-acggagatctatgtcgccggcggcggc-3′ and 5′-gccgccgccggcgacatagatctccgt-3′, K40A used 5′-cctcgccatctttggagcgaccgagggcaatggc-3′ and 5′-gccattgccctcggtcgctccaaagatggcgagg-3′, K156A used 5′-gcatttcgtgcaggtggcatgcccgcttctcacc-3′ and 5′-ggtgagaagcgggcatgccacctgcacgaaatgc-3′, K285A used 5′-catcgtgctcgccgcggcggagcccagc-3′ and 5′-gctgggctccgccgcggcgagcacgatg-3′, R188K used 5′-ctcaaatccatgggcctctcaaagggggcgagcgc-3′ and 5′-gcgctcgccccctttgagaggcccatggatttgag-3′, and R188Q used 5′-tgggcctctcacagggggcgagcgcg-3′ and 5′-cgcgctcgccccctgtgagaggccca-3′. The protein yield of both wild type and mutant enzymes was 7–10 mg/L. CD spectroscopy experiments were conducted over the range of 200–250 nm on a JASCO J-815 CD spectrophotometer equipped with a Peltier temperature control (JASCO Inc.). The wild type and mutant enzymes were analyzed by circular dichroism (CD) and indicated that the proteins had the correct secondary structure and were stable. The proteins were dissolved at a concentration of 14 µM in 0.1 M potassium phosphate buffer, pH 7.0. For far-UV CD data collection, the measurements were carried out in 1 mm path length cell at 25°C. All measurements were an average of three scans. For thermal melting, the changes in CD spectra of the proteins were monitored at 220 nm during the course of heating the samples from 20°C to 90°C. No precipitation or signs of enzyme instability was observed.

### Crystallization

Initial crystallization experiments were previously described [10]. The crystals in this paper were obtained using the hanging drop vapor diffusion method at 20°C. Optimized crystallization conditions suitable for X-ray diffraction experiments contained 1.0–1.7 M magnesium sulfate, 0.1 M Tris-HCl pH 7.0–7.5. Suitable crystals for the X-ray diffraction experiments were observed within three days.

### Structure Determination and Refinement

Crystal structure of the CAH-barbituric acid complex was determined by the single-wavelength anomalous dispersion (SAD) phasing method using a dataset collected at the Se peak wavelength (0.97864 Å) on a selenomethionine-substituted CAH crystal. Details of the X-ray diffraction data collection were reported earlier [10]. Twenty nine selenium atoms were located and phases calculated by PHENIX [12]. Autobuild by RESOLVE [13] built about 60% of the protein residues for two monomers in the asymmetric unit of the crystal. Iterative cycles of model refinement by PHENIX and model building using COOT [14] yielded a final model with R-work of 15.94% and R-free of 19.13% at 2.7 Å resolution. The final model contains two protein chains with 351 and 347 amino acid residues, 3 barbituric acid molecules, 3 Mg^2+^ ions, 10 sulfate ions, and 172 water molecules.

Barbituric acid is not three-fold symmetrical and has a carbon instead of one of the nitrogens in the cyanuric acid substrate (Fig. 1). The electron density readily identifies the position of the barbituric acid ring and the carbonyls within the ring, but the CAH structure resolution was not sufficient enough to immediately determine the position of a carbon versus a nitrogen in the ring. Therefore, the barbituric acid molecule at the active site was modelled in three different orientations, each with 120° rotation around the axis perpendicular to the plane of the ring. Simulated annealing refinement was carried out for each barbituric acid orientation, and difference electron density maps were generated. The three different orientations yielded similar 2Fo-Fc maps but distinctive Fo-Fc maps. The present model yields a clean 2Fo-Fc map without any residual Fo-Fc map peaks, while the other two orientations of barbituric acid yield Fo-Fc maps with positive and negative peaks.

Quality of the model was assessed by PROCHECK [15], and no amino acid residue was found to be in the disallowed region of the Ramachandran plot. The summary of data collection, model refinement, and model quality is shown in Table 1. Atomic coordinates and structure factors have been deposited in the RCSB Protein Data Bank under the accession code 4NQ3.

**Table 1:** Data collection and refinement statistics.

	CAH
Space group	*I4_1_22*
Cell dimensions	
* a, b, c (Å)*	237.9, 237.9, 105.3
Wavelength (Å)	0.97864
Detector	NOIR-1
Resolution range (Å)	50.0–2.70 (2.75–2.70)
*R*_sym_^a^	0.088 (0.566)
R_pim_ [30]	0.035 (0.242)
R_meas_ [31]	0.113 (0.731)
CC1/2 [32]	0.976 (0.870)
CC* [32]	0.994 (0.964)
Completeness (%)	98.4 (94.7)
Redundancy	9.9 (8.2)
I/σ (I)	20.3 (2.20)
Total No. of reflections	403575 (15752)
No. of unique reflections	40849 (1921)
FOM^b^ after Density Mod.	0.69
R_work_	0.1594 (0.2665)
R_free_	0.1913 (0.3228)
Average B-factor	53.40
Macromolecules	53.30
Barbituric acid	
active site (A chain)	26.79
active site (B chain)	42.74
surface-bound	73.62
SO_4_^2−^ and MG^2+^	99.46
Water	43.40
R.m.s.d from ideality	
Bond lengths^c^ (Å) (r.m.s.d^b^)	0.009
Bond angles^c^ (*°*)	1.20
Ramachandran plot (%)	
Most favored	92
Additional allowed	8
Generously allowed	0
Disallowed	0

a = Σ|*I*_obs_—*I*_avg_|/Σ*I*_obs_, (*I*_obs_ stands for the observed intensity of individual reflection, *I*_avg_ is average over symmetry equivalents).

Statistics for the highest resolution shell are shown in parentheses.

b = Figure of merit in phasing.

c = root mean square deviation from idealized geometry.

### Biuret Hydrolase-Coupled Ammonia Detection Assay

A coupled-protein assay was developed as a highly sensitive method to measure CAH activity. CAH and mutant enzymes (25–28 µM) were incubated with 0.5 ml of 10 mM cyanuric acid in 0.1 M potassium phosphate buffer (pH 7) for time periods ranging from 0.5–65.0 h. The CAH reactions were stopped at four discrete time points by boiling the reaction tubes for ten minutes. Next, the reaction tubes were cooled to room temperature, a 5 µg aliquot of purified biuret hydrolase was added to each tube, and then the tubes were incubated at room temperature for 1 h. After incubation, ammonia was quantitated colorimetrically via the Berthelot reaction [16]. Specific activity of the mutant CAHs was calculated at each time point based on 1 mole ammonia/1 mole biuret, and 1 mole biuret/1 mole cyanuric acid cleaved. Control samples without enzyme(s) were incubated in parallel to determine background levels of cyanuric acid hydrolysis or ammonia release. All samples were analyzed in triplicate.

### Sequence Family Analysis

The CAH/barbiturase famly was updated, using methods described previously [7]. As of October 5, 2013, 119 sequences were collected. These sequences were subdivided into ten clades based upon phylogenetic neighbor-joining trees produced by PHYLIP. Consensus sequences were determined for each of the clades, making distinctions between 100% and 50% conservation. Conserved residues were cross-referenced with the structure using the program Chimera [17].

### Structural Analysis

The program LIGPLOT was used to analyze the tetramer interface [18]. The programs Areaimol [19], within the program suite CCP4 [20], and CNS [21] were used to calculate accessible surface areas of amino acid residues and buried surface area.

### Figure Preparation

Pymol was used for figure preparation in the manuscript. (http://pymol.sourceforge.net/)

## Results

### Overall Architecture of CAH

**Figure 2:**
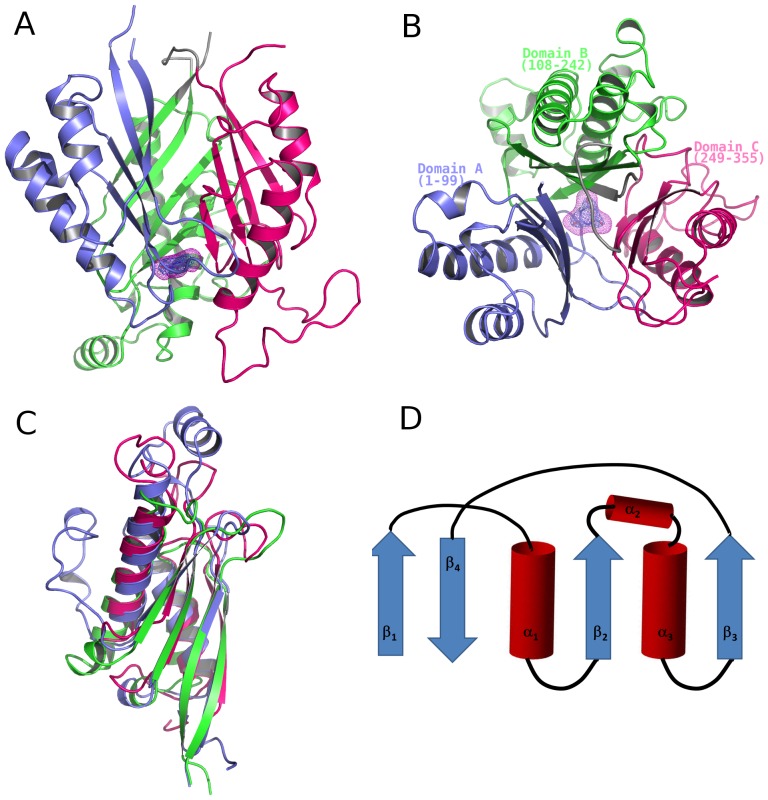
Monomer of CAH. A. The side view of monomer structure. The selenium SAD-phased electron density for the barbituric acid inhibitor is shown in blue mesh, contoured at 2.0 σ. The simulated-annealing omit Fo-Fc map with the barbituric acid inhibitor omitted is shown in magenta mesh, contoured at 3.0 σ. Domains are depicted in different colors: Domain A, consisting of residues 1–99, in blue; Domain B, consisting of residues 108–242, in green; and Domain C, consisting of residues 249–355 in hot pink. B. The top view of monomer structure compared to A, looking through the center of the barrel along the pseudo three-fold axis. C. Overlay of the three domains of CAH. D. Depiction of the trimeric repeat domain fold in CAH. Red cylinders represent α-helices and blue arrows represent β-strands. α-helices 2 and 3 are present in the domain B, while domains A and C have a loop in place of α2.

The crystal structure determination of a selenomethionine substituted CAH-inhibitor complex was accomplished using the single wavelength anomalous diffraction (SAD) phasing. The final 2.7 Å resolution model revealed a protein fold consisting of a β-barrel-like core with α helices organized on the outside, forming a central cavity ∼10 Å wide at its opening. (Fig. 2A–B). At the bottom of the central cavity, the barbituric acid inhibitor is bound.

In the CAH-barbituric acid complex crystal, the asymmetric unit contains a dimer related by a non-crystallographic two-fold axis (Fig. S1A) that further dimerizes via a crystallographic two-fold axis to form a tetramer with 222 point symmetry (Fig. S1B). The CAH tetramer is stabilized by extensive interactions between the monomers, burying a total of 3840 Å^2^ of accessible surface area which is 12.41% of the total surface area. The molecular interface consists of a large number of polar interactions, particularly in the core of the tetramer, involving a total of 88 residues forming 58 hydrogen bonds (counted with a distance cutoff of 2.5∼3.2 Å) as well as several aromatic residues making hydrophobic interactions (Fig. S2). The formation of the CAH tetramer in the crystal is consistent with our earlier observation by size-exclusion chromatography [10].

The structure of the CAH monomer exhibits an internal pseudo-three fold symmetry, consistent with our preliminary analyses of the X-ray diffraction data (Fig. 2B) [10]. Structural similarity is evident between three separate sections of the CAH monomer: domain A, 1–99 (Fig. 2, blue); domain B, 108–242 (Fig. 2, magenta); and domain C, 249–355 (Fig. 2, cyan). The three domains are superimposable with root mean square deviations (RMSD) of alpha carbons of 3.2 Å, 2.0 Å, and 2.2 Å for domains A and B, domains A and C, and domains B and C, respectively (Fig. 2C), though protein sequence identity among these three domains is limited to 13–17%. Each domain of the protein has a conserved fold consisting of a 4-stranded β-sheet, and either 2 or 3 α-helices, in the topology depicted in Fig. 2D.

### Comparison of CAH to AtzD and Evolutionary Links to Other Protein Families

**Figure 3:**
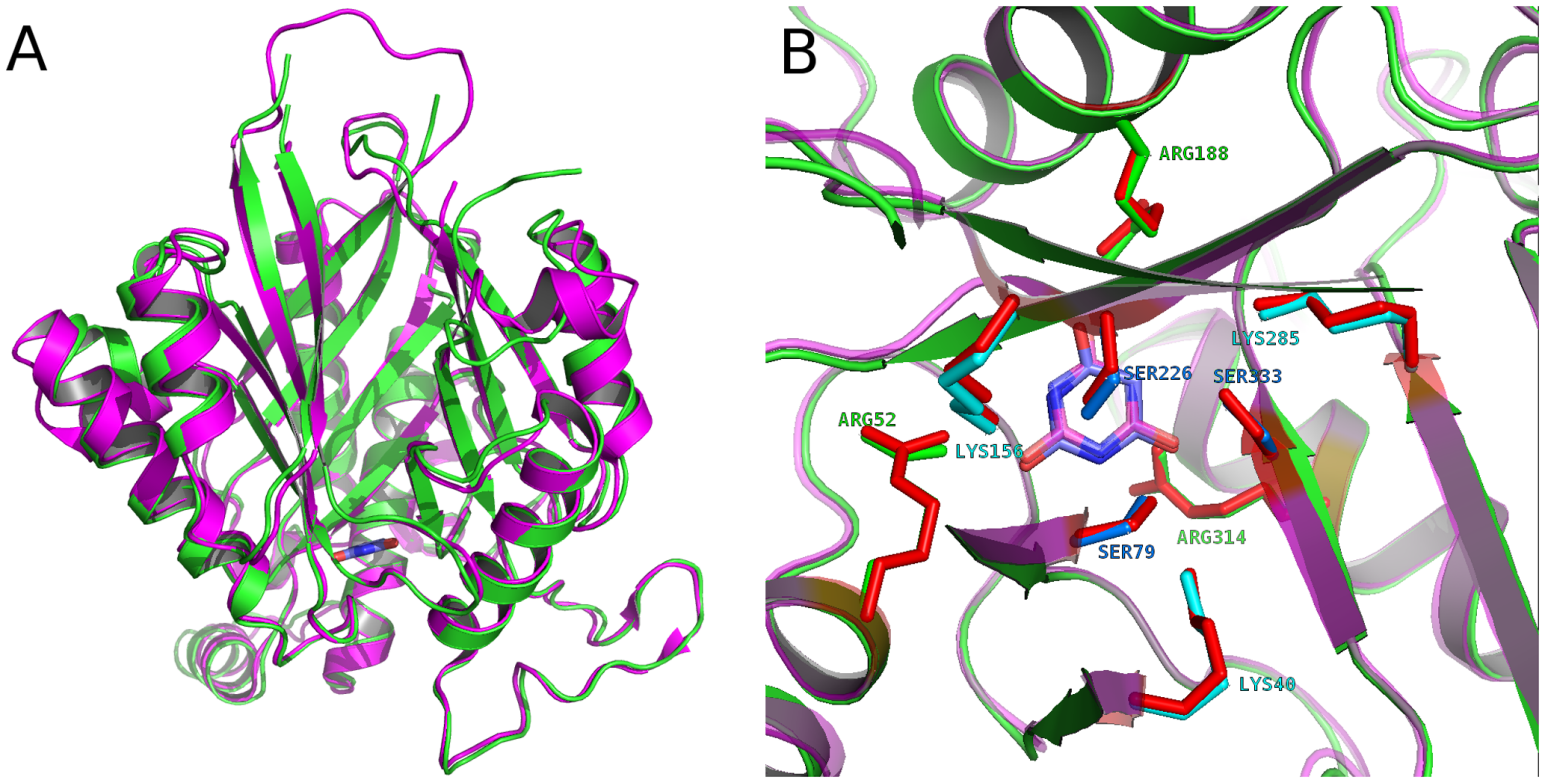
Structural comparison between CAH and AtzD. A. Comparison of overall structures between CAH and AtzD monomers. CAH is colored in green while AtzD is colored in magenta. B. Comparison of the active sites between CAH and AtzD showing highly similar arrangement of the residues involved in substrate binding and catalysis. Residue labels are for CAH.

The pseudo three-fold symmetrical protein fold of *Azorhizobium* CAH has been recently described as the Toblerone or tricorne fold for AtzD from *Pseudomonas sp*. stain ADP, another member of the CAH/barbiturase protein family [11]. These proteins share 51% sequence identity and differ in their enzyme kinetic values. Though *k*_cat_ values of both enzymes are close (AtzD: (73±6) s^−1^ and *Azorhizobium* CAH (50±9) s^−1^), there is an order of magnitude difference for *k*_cat_/*K_m_* values, (AtzD: (3.2±1.2)×10^6^ s^−1^ M^−1^ and CAH: (1.3±0.6)×10^5^ s^−1^ M^−1^), due to a ∼10-fold differences in their *K_m_* values (AtzD: (23±7) µM and CAH: (370±90) µM (7). The difference in *K_m_* values implies that there may be differences affecting substrate-protein interactions, despite similar structures (RMSD for 334 Cα atoms, 0.86 Å) (Fig. 3A) with highly similar active site residues (Fig. 3B), which suggests a common catalytic mechanism.

The residues positioned within 5 Å of the barbituric acid inhibitor are, in fact, identical between the two proteins except for Ser227 in CAH corresponding to Ala233 in AtzD (4BVR). The backbone nitrogens of these differing residues are 2.64 Å and 3.01 Å from a substrate carbonyl, respectively, and the backbone carbonyls are 2.82 Å and 2.71 Å from a substrate ring nitrogen. Ser227(CAH) and Ala233(AtzD) may, therefore, assist in substrate binding. This single residue difference is therefore not expected to alter substrate binding to the degree observed. Remote mutations are also known to affect substrate binding [22], [23], making more study into *K_m_* differences between these two proteins necessary.

**Figure 4:**
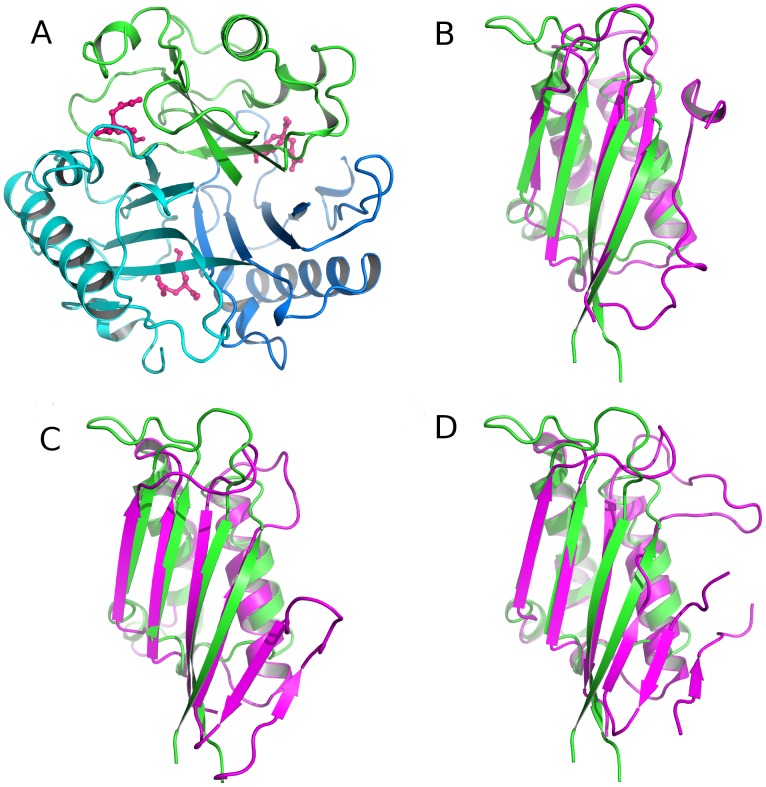
Comparison between CAH fold and the homotrimeric proteins in the YjgF-like SCOP structural superfamily. A. The trimer of AroH-type chorismate mutase (CM) with active sites in each subunit interface that is accessed from the exterior of the barrel. B, C and D show the overlays of CAH domain A (residues 1–99, green) and a monomer of various structurally similar homotrimeric proteins (magenta): B. AroH-type CM (1COM). C. Proposed translation initiation inhibitor (PDB ID: 3GTZ). D. Putative aminoacrylate peracid reductase, RutC (PDB ID: 3V4D).

The DALI server [24] was used to identify proteins with folds similar to that of *Azorhizobium* CAH. Besides AtzD, which is closely related to *Azorhizobium* CAH structurally and functionally, the best scoring protein was the AroH-type chorismate mutase (CM) (PDB ID: 1XHO) (Fig. 4A) with a Z-value of 7.8. This protein belongs to the YjgF-like SCOP structural superfamily [25], [26], [27], which contains homotrimeric proteins with active sites located at each of the subunit interfaces. These active sites are on the external face of the barrel (Fig. 4A), and the internal cavity or the head of the barrel is not known to have a catalytic function. This contrasts with CAH, which is a single polypeptide with a single active site on the inside of the barrel structure. Overlays of the individual domains of CAH on AroH-type CM (Fig. 4B) yield an average RMSD of 2.88 Å, despite only ∼9% sequence identity. The difference in the active site arrangements and low protein sequence identities between CAH and the YjgF-like superfamily proteins make it unclear whether this similarity is due to convergent or divergent evolution.

### Active Site Architecture of CAH

In both subunits in the asymmetric unit of the CAH-barbituric acid complex crystal, a molecule of the highly analogous substrate analog, barbituric acid was found at one end of the barrel structure, unambiguously identifying the location of the active site and the mode of substrate-binding (Fig. 2B). In addition, another barbituric acid molecule, albeit at lower occupancy, was observed on the surface of one of the CAH subunits near the opening of the potential substrate channel, which is discussed later in more detail.

**Figure 5:**
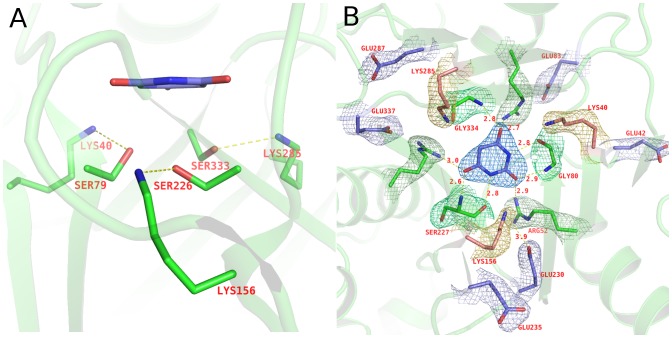
Trimeric conglomeration of CAH active site. A. Three serine residues (Ser79, Ser226, Ser333) lie within hydrogen bond distances from corresponding lysine residues (Lys 40, Lys 156, and Lys 285, respectively). Each pair shows the typical arrangement for the Ser/Lys dyad hydrolase. B. Three arginine residues (Arg 52, Arg 188, Arg 314) and the backbone nitrogens from Gly80, Ser227 and Gly334 form hydrogen bonds with the carbonyl oxygens of the barbituric acid inhibitor. The simulated annealing composite-omit 2Fo-Fc map is shown in colored mesh, contoured at1.5 σ.

The three-fold structural symmetry of CAH extends to the arrangement of residues that compose the active site. A highly organized, trimeric conglomeration of serines, lysines, and arginines surrounds the bound barbituric acid (Fig. 3B), mirroring the three-fold symmetrical shape of the substrate. Three serine residues (Ser79, Ser226, Ser333) lie in close proximity to the carbonyl carbons of barbituric acid, suggesting that one could act as the nucleophile in a nucleophilic attack on the substrate. These residues are analogous to the three serines identified in AtzD [11]. Because the serines are positioned below the plane of the barbituric acid ring and the bound inhibitor is not exactly centered in the active site, each of the three serines are at various distances from the carbonyl carbons within the bound barbituric acid (Fig. 5B). Ser79 γO has distances of 3.65 Å, 4.66 Å, and 3.21 Å from the carbonyl carbons facing domains A, B, and C, respectively. Likewise, Ser226 γO is 3.65 Å, 3.57 Å, and 4.16 Å; and Ser333 γO is 5.59 Å, 4.71 Å, and 3.73 Å, to those same domain-facing carbonyl carbons, respectively.

Measurement of Bürgi–Dunitz (BD) angles was used to obtain greater insights into the residues potentially involved in nucleophilic attack on the substrate. The BD angle is the angle that defines the geometry between a nucleophile and a trigonal unsaturated center like a carbonyl carbon, with acceptable BD angles of 105±5° [28]. The BD angles for Ser226 attacking either of two carbonyl carbons in the bound inhibitor were within reasonable values (105.8° for the carbonyl facing domain A and 102.6° for the carbonyl facing domain B). In contrast, only one BD angle was reasonable for Ser79 (103.4° for the carbonyl facing domain A) or Ser333 (110.0° for the carbonyl facing domain B). Combining distances and BD angle data, it appears that Ser79 has an unreasonable BD angle for the carbonyl carbon facing domain C, despite the close distance. This was proposed to be the catalytic serine and carbonyl that is attacked for AtzD [11]. The carbonyl carbon with the best BD angle for Ser79 has a distance (3.65 Å) which is a bit far unless there is some conformational change. Ser226 has the domain A and B-facing carbonyl carbons with reasonable BD angles and distances. Ser333, however, has a very long distance (4.71 Å) for the carbonyl with the only BD angle within reasonable parameters. The combination of distance and BD angles data indicate that Ser226 is the most likely nucleophile of the three serines in the CAH active site.

As seen with AtzD, each serine is accompanied by a lysine residue (Lys40, Lys 156, and Lys 285, respectively for Ser79, Ser226, and Ser333), positioned within hydrogen bonding distances (Fig. 5A). These lysines are positioned optimally to activate the serine residues. The guanidinium group of three arginine residues (Arg 52, Arg 188, Arg 314), one from each domain, and the backbone amide nitrogens of the residues following the active site serines (Gly80, Ser227 and Gly334) are within 3.0 Å from the three carbonyl oxygens of the bound barbituric acid (Fig. 5B), demonstrating potential protein-substrate interactions which could be involved in substrate activation and be the oxyanion hole for the carbonyl that undergoes nucleophilic attack.

### Catalytic Properties of Mutants

**Figure 6:**
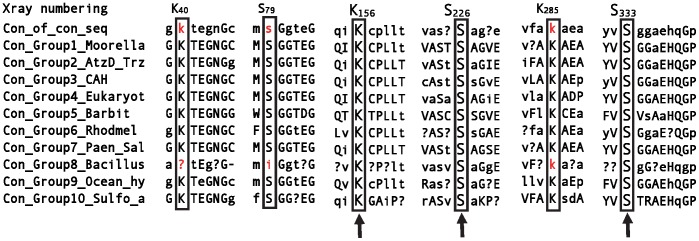
Sequence alignment showing conservation of three Ser/Lys dyads found within subgroups of the CAH/barbiturase family. Subgroups of the updated family of 119 sequences were determined via phylogenetic clades. Consensus sequences were determined for each of the subgroups. Only sequences directly surrounding the dyads are displayed. Absolute, 100% conservation is represented by capital letters and greater than 50% conservation by lowercase letters. Columns in which no single residue was conserved at greater than 50% are depicted by a question mark. This usually occurred in regions of highly divergent sequence or where subgroups within a clade contained different residues. Residues equivalent to the Ser/Lys dyad residues are outlined with black boxes. Arrows point to Ser/Lys dyad residues with complete conservation across the family; residues not conserved in these positions are highlighted in red.

To obtain further insights into catalytic roles of the active site residues, site-directed mutagenesis was performed. Development of a sensitive, coupled enzyme assay in this study specifically allowed for determination of activities that were not possible in previous studies. All of the Ser to Ala mutants (S79A, S226A, S333A) exhibited reduced activities with cyanuric acid compared to the wild-type enzyme (Table 2). The S226A mutant showed the greatest decrease, approximately 109-fold. The activities of S79A and S333A were 108-fold lower than that of wild type. These results indicate that all three serines play roles in CAH catalysis. The 20–40 fold greater effect of the S226A mutant is consistent with the possibility of this serine serving as a nucleophile. Moreover, when analyzing the primary sequences of all homologs within the CAH/barbiturase family, only one Ser/Lys dyad is absolutely conserved and corresponds to the Ser226/Lys156 dyad in CAH (Fig. 6). In total, the results suggest that Ser226 acts as a nucleophile and that the other two serines are involved in binding and activation of the substrate.

**Table 2:** Specific activity of wild type and mutant CAH on cyanuric acid as determined by the biuret hydrolase-coupled ammonia detection assay*.

	Specific activity
	(µmol/min per mg)
Wild type	11.5±3.5
S79A	(6.0±1.3)×10^−7^
S226A	(2.7±0.4)×10^−8^
S333A	(1.7±0.4)×10^−7^
K40A	(8.0±2.8)×10^−4^
K156A	(1.0±0.2)×10^−3^
K285A	(1.4±0.1)×10^−3^
R188K	(1.5±0.1)×10^−3^
R188Q	(3.2±0.5)×10^−4^

*Results were performed in triplicate.

Mutation of any of the three active site lysine residues within hydrogen bonding distance to the active site serines (Lys 40, Lys 156, and Lys 285) causes ∼104-fold reduction of specific activity (Table 2). One of these lysine residues could act as a base to activate the serine nucleophile (Fig. 3B). The similarity between CAH and Ser-Lys serine proteases [29] is currently unclear, but it is interesting that CAH is not inhibited by classical serine protease inhibitors like PMSF (data not shown) similar to many of the Ser-Lys dyad proteins. This differs from the AtzD enzyme in which inhibition was observed upon treatment with PMSF [11]. Aside from the residues mentioned above, there are no other ionizable groups within 8 Å of the three serines except for Asp49 and Tyr331. The side chain oxygens (δO1 and δO2) of Asp49 are 7.5–12.5 Å remote from the γO of the active site serines and Tyr331 side chain oxygen at 4.4 Å is 1.5 Å further away from the Ser333 γO than the nitrogen side chain of the corresponding Lys285 making either residue a less likely candidate for a general base catalyst.

**Figure 7:**
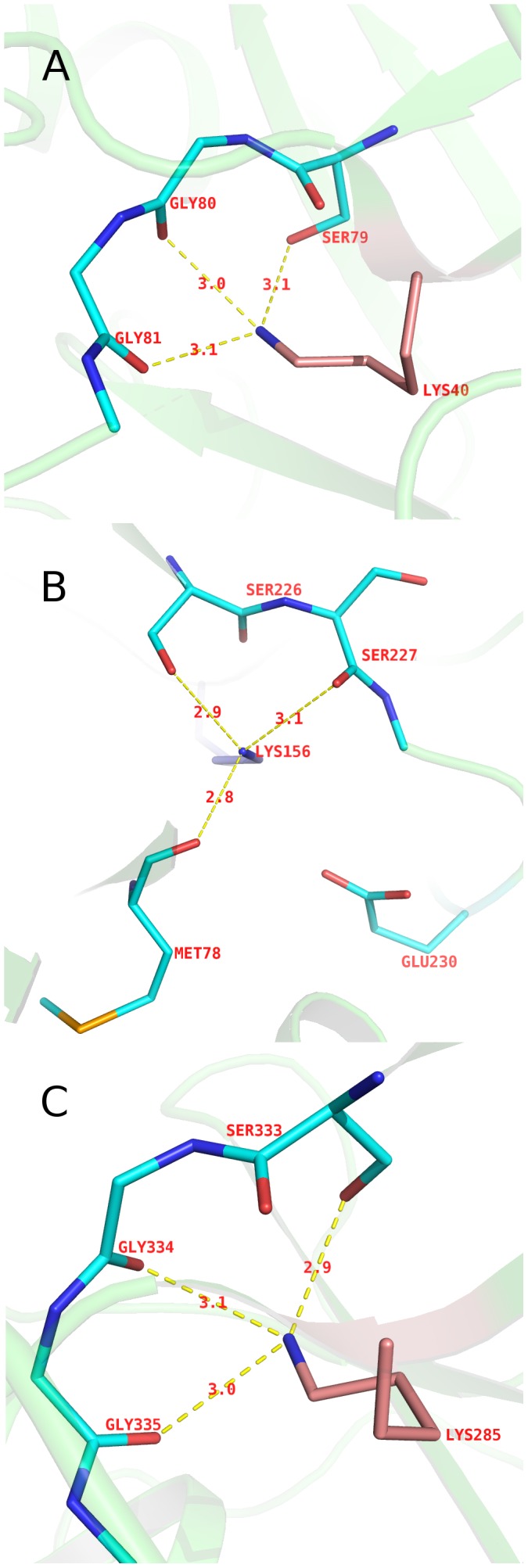
Hydrogen bonding and electrostatic interactions network around three lysines from each domain in CAH. A. Lys40 forms hydrogen bonds with the backbone carbonyls of Gly80 and Gly81, and the Ser79 side-chain. B. Lys156 is hydrogen-bonded to backbone carbonyls of Met78 and Ser227, and Ser226 side-chain. In addition, Glu230 forms electrostatic interaction with Lys156. C. Lys285 is hydrogen bonded to backbone carbonyls of Gly334 and Gly335, and Ser333 side-chain.

To further assess the potential catalytic roles of these Lys residues, we examined their environments. All three lysines have extensive hydrogen bonding to backbone carbonyl groups in addition to respective serine residues. Lys40 is hydrogen-bonded to Gly80 and Gly81; Lys156 with Met78, and Ser227; and Lys285 with Gly334 and Gly335 (Fig. 7). Furthermore, these lysine residues are completely buried in the interior of the protein, with the accessible surface area (ASA) for the ε-amino group of 0 Å^2^ compared with the average ASA of other lysine residues in the CAH structure of 30.3 Å^2^. This likely is responsible for adjusting the pKa of the lysine residues for catalysis.

Three arginine residues (Arg52, Arg188, Arg314) were within 3.0 Å of the substrate analog's carbonyl oxygens. To investigate the potential role of these arginines in catalysis, two mutants were created (R188K, R188Q). We measured the specific activities of two mutants with the biuret hydrolase-coupled ammonia detection assay and found that R188K and R188Q showed 5,000 and 23,000 fold reductions of specific activities respectively compared to the wild type (Table 2). The greater loss of activity in the R188Q mutant could indicate that the positive charge on the arginine is important for catalysis. The arginines could be involved in substrate binding by interactions with the carbonyl oxygens of the substrate and act as part of the oxyanion hole that stabilizes that charge on tetrahedral intermediates.

### Proposed Catalytic Mechanism of CAH

The active site of CAH with the pseudo 3-fold rotational symmetry constructed from 3 structurally homologous domains seems optimal for catalyzing hydrolysis of the 3-fold symmetrical cyanuric acid. The classical serine hydrolases with the Ser-His-Asp catalytic triad, such as trypsin, initiate catalysis using a negatively charged oxygen atom of serine as a nucleophile. This negatively charged serine oxygen is formed by deprotonation of the gamma hydroxyl group by the catalytic histidine. By stabilizing a positively charged imidazole-ring of the histidine, the aspartate improves the base property of the histidine. Due to the absence of histidine and aspartate residues in the CAH active site and the different chemical environment of the Ser-Lys pair, the same enzymatic mechanism is not possible in the CAH.

**Figure 8:**
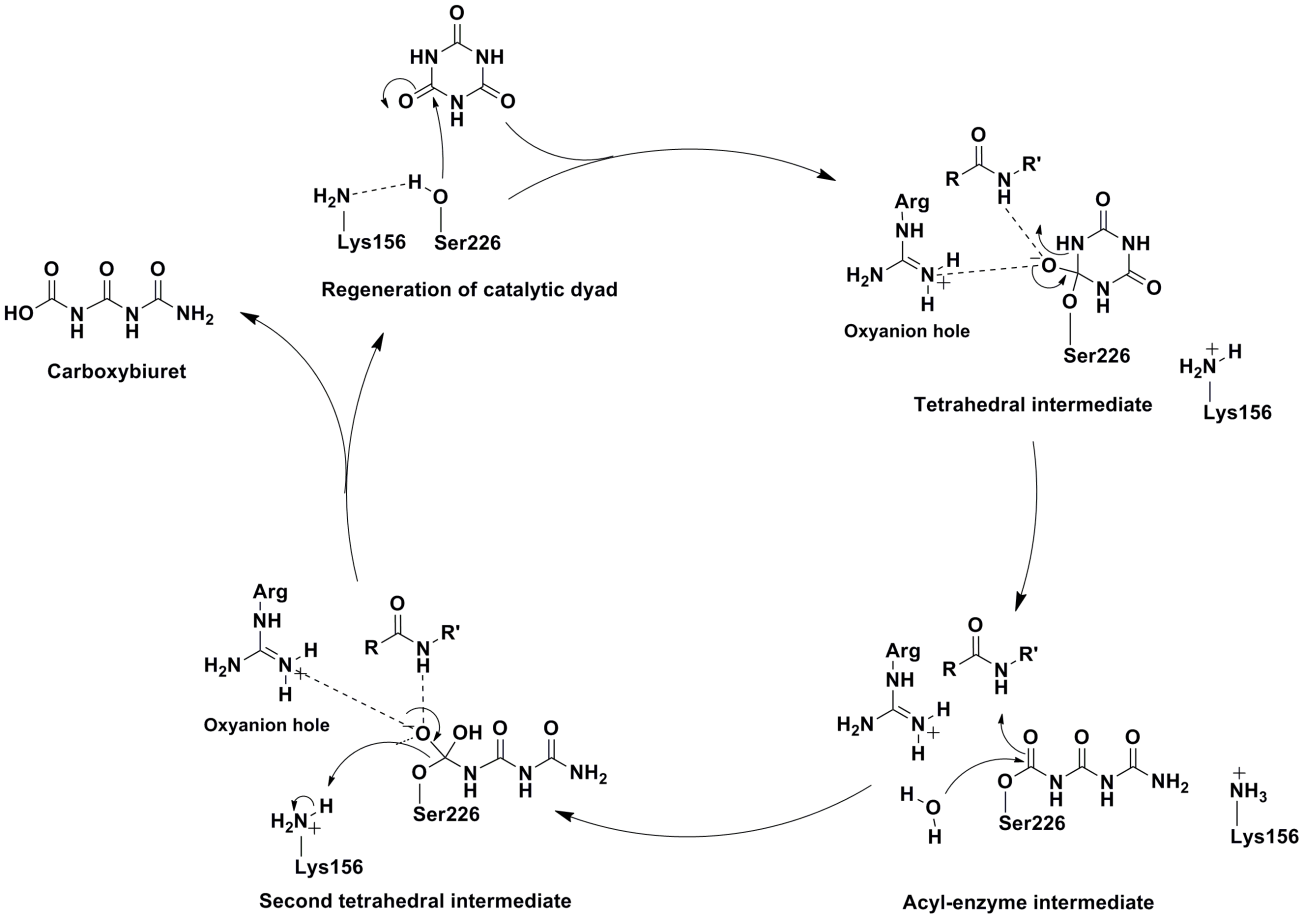
Proposed mechanism for CAH. After substrate binding, Ser226 attacks the substrate carbonyl, producing an tetrahedral intermediate. Arginine and a backbone amide group form an oxyanion hole to stabilize the negative charge on intermediates. The substrate carbonyl reforms with the cleavage of the C-N ring bond. Water then attacks the acyl intermediate to release the product.

Based on the results described above suggesting that Ser226 is the nucleophile, the following reaction steps could be envisioned (Fig 8). Lys156 deprotonates the gamma oxygen of Ser226 to facilitate nucleophilic attack on one of the substrate's carbonyl carbons. The first tetrahedral intermediate would ensue and the negative charge that results is stabilized by an oxyanion hole composed of an arginine and backbone amide. As the carbonyl reforms, the ring C-N bond is cleaved. A water molecule then attacks the acyl-enzyme intermediate to regenerate the resting enzyme after product release.

### Surface-Bound Barbituric Acid and Channels to the Active Site

**Figure 9:**
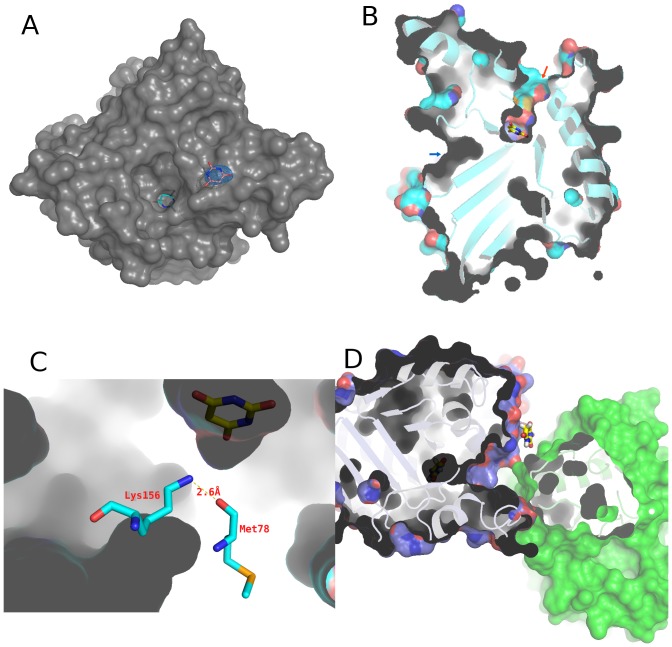
Front and back channels around the active site of CAH. A. A view looking along the front channel down to the active site-bound barbituric acid. A second barbituric acid molecule, found on the surface is also shown in sticks with a simulated annealing composite omit 2Fo-Fc map contoured at 2.0 σ. B. Cut-away view of the CAH monomer. Barbituric acid is seen at the base of the front-side channel (red arrow). The back channel (blue arrow) is blocked by two hydrogen-bonds formed by Lys156 and Met78 (shown in sticks), and by Lys156 and Ser226, and therefore does not reach the substrate-binding site. C. Close-up view of the hydrogen bonds between Lys and Met78. D. Barbituric acid molecule bound near the entry of the front side channel, on the tetramer interface cleft.

There are two entries (“front” and “back” doors) to the active site of CAH (Fig. 9A–B). The central cavity (front door, highlighted by a red arrow) has a funnel-like shape and runs down to the active site along the pseudo 3-fold axis. This channel likely serves as the path for the substrate/product to diffuse in and out. The channel has a diameter of ∼10 Å at its mouth and ∼4 Å at the narrowest point, the latter of which is smaller than the size of the substrate, cyanuric acid. Therefore, some conformational changes would be required for the residues lining the wall of the cavity to allow passage of cyanuric acid. In addition, the opening of this channel to the solvent is partially blocked due to protein tetramerization (Fig. 9D), which limits the access of the entering substrate. In this regard, it is notable that, for one of the molecules in the asymmetric unit, a barbituric acid molecule is found bound on the protein surface near the opening of the central channel (Fig. 9A, 9D). This non-active site barbituric acid molecule bound near the protein-protein interface might provide insights into how the substrate is attracted to the long and narrow tunnel leading to the active site.

The cut-away view of CAH (Fig. 9B) shows that it also has the “back door” between domains A and B, similar to that seen in AtzD [11]. This back-tunnel (highlighted by a blue arrow in Fig. 9B) is blocked by Lys156 that is hydrogen-bonded to the main-chain carbonyl oxygen of Met78 and the Ser226 side-chain, therefore is not reaching the active site (Fig. 9B, 9C). The AtzD structure has an equivalent residue Lys162, hydrogen bonded to Met84 [11]. It is unclear whether this tunnel in the back has any role in substrate/product passage.

## Discussion

The reported crystal structure of CAH has internal three-fold symmetry, originating from the concatenation of three homologous domains. Each domain contributes equivalent active site residues, producing a three-fold symmetry within the active site. The symmetry of the cyanuric acid substrate extends this theme to the active site's substrate binding. Geometry and substrate analog positioning within the active site indicates that CAH likely utilizes a Ser-Lys dyad for catalysis. However, as there are three Ser-Lys dyads in the active site, additional analyses were required to address their specific roles.

With the highly sensitive biuret hydrolase-coupled ammonia detection assay, the catalyticcontribution of each serine to enzyme catalysis could be determined, in contrast to the study with AtzD in which no activity could be detected for any of the serine mutants. The greater loss of activity from the Ser226 mutant in the present study suggested that this could be a catalytic nucleophile. This assignment was further supported by sequence alignments between the cyanuric acid hydrolase/barbiturase family members which revealed that only the Ser226-Lys156 dyad was absolutely conserved across the entire family.

We have proposed that Lys156 serves as the general base to deprotonate Ser226 for a nucleophilic attack on cyanuric acid. The side-chain of lysine is typically protonated at physiological pH (around 7) due to its relatively high pKa (∼10). This would not be compatible with the proposed role for Lys156 as a base. The crystal structure, however, revealed that Lys156 is in a unique microenvironment compared to other lysines in CAH. First, as mentioned above, the ε-amino groups of the three active site lysines (Lys40, Lys156, Lys285) are buried in the interior of the protein. When substrate binds to the active site, the ε-amino groups become completely inaccessible to solvent. Second, the hydrogen-bonding and electrostatic interactions involving Lys156 specifically provide it with a unique environment amongst the three lysine residues in the active site. The ε-amino group of Lys156 is located within ∼3 Å from three oxygen atoms (side-chain hydroxyl group of Ser226 and main-chain carbonyl groups of Ser227 and Met78) as well as ∼3.9 Å from the side chain carboxyl group of Glu230 (Fig. 7B).

There are two glutamic acids around each of the three lysines from each domain (Glu42 and Glu83 around Lys40, Glu230 and Glu235 around Lys156, Glu287 and Glu337 around Lys285) within a distance of 6 Å, expanding the three-fold symmetrical arrangement of protein residues surrounding the active site (Fig. 5B). Only Glu230 is absolutely conserved amongst the AtzD/barbiturase family members. This residue is within a distance to allow direct ionic interaction with Lys156 (∼3.9 Å). This would indicate that the Ser-Lys dyad may in fact be a Ser-Lys-Glu active site with Glu230 stabilizing the positive charge on Lys156 after it takes the proton and activates the gamma oxygen of Ser226. This configuration of active site residues has not, to our knowledge, been identified in the literature, but mirrors the other assortment of residues used to activate a serine nucleophile within serine hydrolytic enzymes [29].

The active site of AtzD from *Pseudomonas sp*. stain ADP [11] also consists of three pairs of Lys/Ser dyads (Lys42/Ser85, Lys162/Ser233, and Lys296/Ser344) with an arrangement that mirrors that of CAH (Fig. 3). Peat et al. proposed that Ser85 of AtzD is acting as a nucleophile in the catalytic cycle. Ser85 of AtzD corresponds to Ser79 from the domain A of CAH. However, Peat, et al. were unable to quantitate activity for any of the three serine mutants. With the additional data from the mutational studies provided by the current study, the evidence favors Ser226 of the *Azorhizobium* CAH as the nucleophile. This residue corresponds to Ser233 of AtzD (Fig. 3B).

Mutational studies also indicated Arg188 to have a role in catalysis. Here, it is proposed that the triplicate arginines (Arg52, Arg188, Arg314) are involved with substrate binding, providing a positive charge to interact with the three carbonyl oxygens. The arginine associated with the carbonyl that is attacked by the nucleophile would also be part of the oxyanion hole that neutralizes the negative charge that forms on the oxygen of tetrahedral intermediates. Family analysis shows that Arg188 is conserved in all except two subgroups. The *Bacillus* subgroup has a lysine, and the barbiturase subgroup has a glutamine. Barbiturase uses barbituric acid as a substrate. Though this compound is analogous to cyanuric acid, it has a carbon in the place of one of the ring nitrogens. This difference results in only one of the carbonyl groups being surrounded on both sides by ring nitrogens, making it asymmetric. This difference could alter the substrate binding requirements for the enzyme and allow a glutamine in this position. Because the glutamine is less likely to stabilize the negative charge of the tetrahedral intermediate than an arginine, it is unlikely that the carbonyl near this residue (facing domain B) is the site of nucleophilic attack by the catalytic serine, at least in the case of barbiturase. The only absolutely conserved arginine is Arg52, making the carbonyl by this residue (in domain A) the most likely site for nucleophilic attack for the whole family.

In one of the CAH molecules in the asymmetric unit, a water molecule (A #509) was found within hydrogen bonding distance (2.99 Å) of the terminal side chain nitrogens of Arg188 This water is positioned 5.15 Å from one of the carbonyl carbons of the bound inhibitor and on the opposite face of the ring compared to the serine nucleophile. Another water (A #609; occupancy of 1 and a B-factor of 45.54, a value comparable to nearby water molecules) is located directly above the barbituric acid ring in a pocket within 2.4–3.1 Å from the OG1 of Thr310, the NH1 of Arg314, and water (A #509). It is 4.2–5.0 Å from the carbonyl carbons of the substrate ring. With either of these waters, some conformational change that reduces the distance to one of the carbonyl carbons would be required for involvement in catalysis, but more studies are required to confirm the function of specific waters in the crystal structure or the exact role of the arginine residues.

The structure and proposed mechanism presented here will serve as a framework for better characterizing the catalytic mechanism of CAH and engineering CAH for improved catalytic properties. Since cyanuric acid is a product of pool water chlorination, a CAH mutant with enhanced activity would be industrially useful for bioremediation and water conservation efforts. Developing an *in situ* swimming pool water treatment system would prevent the need for complete water exchange to remove excess cyanuric acid in pool water. Based on the current CAH structural data and gel-filtration analysis [10], the biological unit of CAH is a tetramer (Fig. S1B). AtzD was also reported to form a stable homotetramer, based on the X-ray crystallographic and SAXS analyses [11]. Although tetramerization could be contributing to the stability of CAH, it does not seem to provide any benefit in enzyme catalysis and rather appears to partially block the substrate entry pathway. Therefore, dissociation of CAH tetramer into monomers or dimers by modifying the molecular interfaces could potentially allow more efficient substrate diffusion to the active site and increase the catalytic turnover of the enzyme.

## Supporting Information

Figure S1 **The oligomerization of CAH.** A. Dimer of CAH found in the asymmetric unit of the crystal. The two molecules are related by a non-crystallographic two-fold axis. The two protein chains are colored green and cyan. B. Tetramer of CAH. The CAH dimer shown in (A) further dimerizes to form a tetramer through a crystallographic two-fold axis. The barbituric acid molecule found on the protein surface is shown in sticks.
(TIF)

Figure S2 **Interactions in tetramer interface.** The panel framed in blue is showing the abundance of polar interactions (hydrogen bonds and salt bridges) in the core of the tetramer. The other two panels (red and black) are showing the presence of both polar and hydrophobic contacts stabilizing different dimeric interfaces.
(TIF)
