# Supplementary figures and images for: Cyanuric Acid Hydrolase from Azorhizobium caulinodans ORS 571: Crystal Structure and Insights into a New Class of Ser-Lys Dyad Proteins
Source: PLoS One. 2014 Jun 10;9(6):e99349. doi: 10.1371/journal.pone.0099349 (PMC4051656; doi:10.1371/journal.pone.0099349)

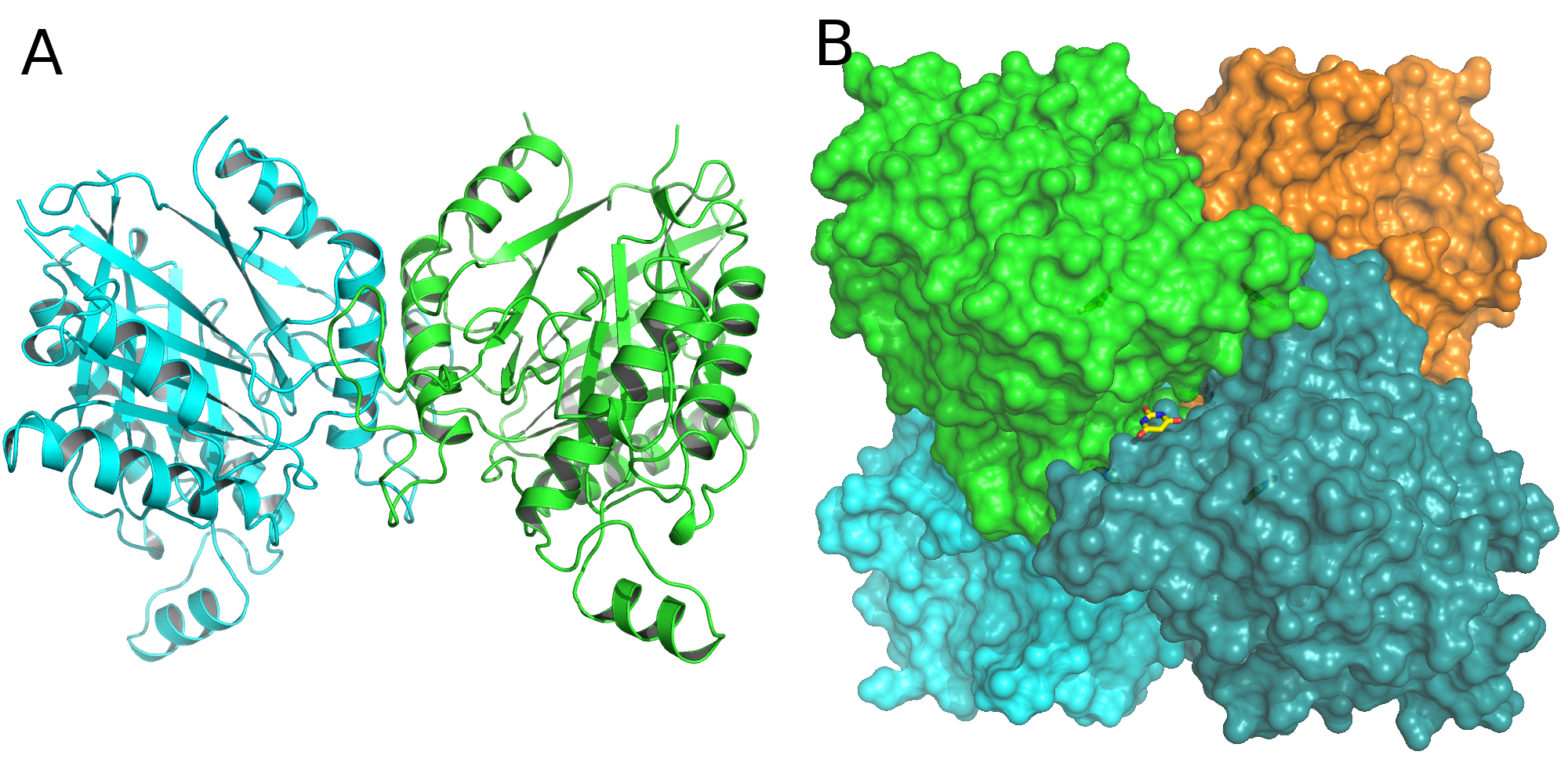

Supplement: Figure S1 — The oligomerization of CAH. A. Dimer of CAH found in the asymmetric unit of the crystal. The two molecules are related by a non-crystallographic two-fold axis. The two protein chains are colored green and cyan. B. Tetramer of CAH. The CAH dimer shown in (A) further dimerizes to form a tetramer through a crystallographic two-fold axis. The barbituric acid molecule found on the protein surface is shown in sticks. (TIF) [file pone.0099349.s001.tif]

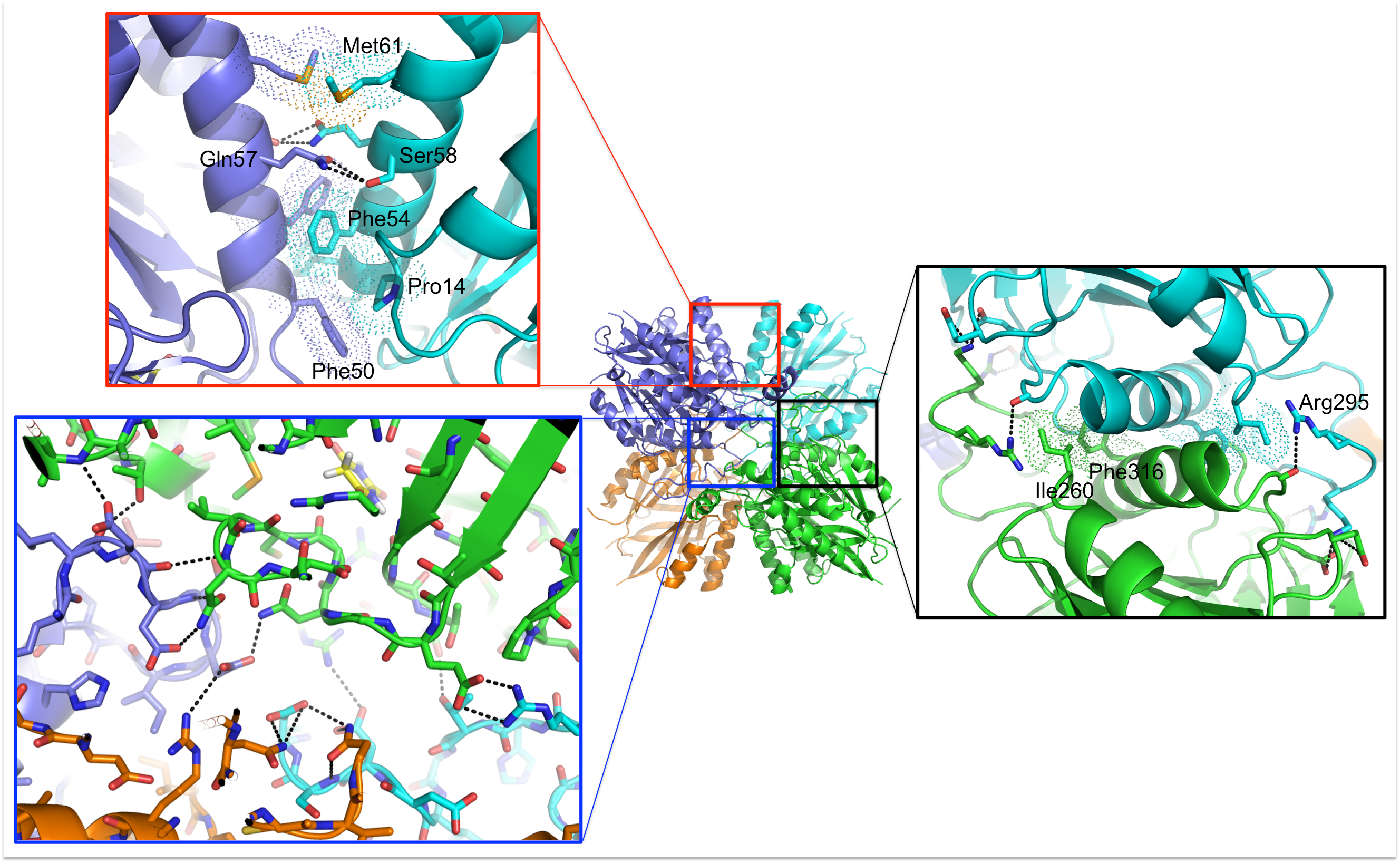

Supplement: Figure S2 — Interactions in tetramer interface. The panel framed in blue is showing the abundance of polar interactions (hydrogen bonds and salt bridges) in the core of the tetramer. The other two panels (red and black) are showing the presence of both polar and hydrophobic contacts stabilizing different dimeric interfaces. (TIF) [file pone.0099349.s002.tif]
